# Reflexion as the Factor in Shaping Attitudes Towards Love and Sex at Adolescence

**DOI:** 10.1192/j.eurpsy.2022.1754

**Published:** 2022-09-01

**Authors:** M. Alyeva, T. Sadovnikova

**Affiliations:** Moscow State University, Faculty Of Psychology, Moscow, Russian Federation

## Abstract

**Introduction:**

Вuilding psychologically close relationships with the partner is an important task of development in adolescence. Dysfunctional relationships is a source of stress, can lead to mental disorder. Reflection is a mental (rational) process aimed at analyzing, understanding, realizing oneself: one’s own actions, behavior, speech, experience, feelings, states; reflexion is a condition of orientation in interpersonal relations, formation of attitudes, perceptions, values, refinement and formation of self and partner image.

**Objectives:**

The aim was to study the reflection role in of the romantic relationship attitudes formation at adolescence.

**Methods:**

The techniques were completed by 84 students 17-21 age (M = 19.23; SD=1,21; M/F= 83.3 / 16.7 %) 1. Attitudes About Love and Sex by C. Hendrick and S. Hendrick (Ekimchik, 2007) 2. Differential Test of Reflection (Osin, Leontiev, 2014) 3. Reflexivity Questionnaire (Karpov, 2004).

**Results:**

The dominant types of reflection are «Systemic reflection» and «Reflexivity of future activity» (A.V. Karpov), which correlates with age-related developmental tasks. «Reflexivity of communication and interaction with others» is found at a rather low level. Cluster analysis highlighted three groups of respondents with different types of reflection: «reflexive» (40 %), «dreamers» (36 %), «communicative» (24 %). The most pronounced types of love are Eros and Agape. There are significant differences in the expression of the love style in three groups of respondents (H-criterion Kraskel-Wallis).

**Conclusions:**

Reflexion features are a factor in the formation of the cognitive-behavioral component of a love in adolescence. Optimal type of reflexion creates conditions for prevention of destructive relationships in youth.

**Disclosure:**

No significant relationships.

